# DNA methylation clock in bull sperm cells reveals the epigenetic aging characteristics and impact on fertility

**DOI:** 10.1186/s40104-026-01444-5

**Published:** 2026-07-02

**Authors:** Wenlong Li, Yongjie Tang, Songyan An, Jiahao Wang, Xia Feng, Siqian Chen, Ying Yu

**Affiliations:** National Engineering Laboratory for Animal Breeding, State Key Laboratory of Animal Biotech Breeding, Key Laboratory of Animal Genetics, Breeding and Reproduction of Ministry of Agriculture and Rural Affairs, College of Animal Science and Technology, China Agricultural University, Beijing, 100193 China

**Keywords:** Bull sperm cells, DNA methylation clock, Paternal aging, Semen quality

## Abstract

**Background:**

Aging is a crucial factor influencing semen quality and fertility in bulls, with potential implications for reproductive management and genetic improvement programs. Understanding the molecular mechanisms underlying sperm cell aging is essential for assessing reproductive potential and improving livestock productivity.

**Results:**

In this study, we developed an epigenetic clock for bull sperm cells using whole-genome bisulfite sequencing (WGBS) and reduced representation bisulfite sequencing (RRBS) on samples from Holstein stud bulls of different ages. Our analysis identified age-specific CpG sites and revealed distinct DNA methylation patterns associated with different age stages. The developed epigenetic clock demonstrated high accuracy in predicting the biological age of bull sperm cells, with significant correlations between epigenetic age acceleration (EAA) and semen quality traits such as fresh semen motility, frozen semen abnormality rate, and testicular circumference. Furthermore, we explored the involvement of transmembrane transport and other pathways in sperm cell aging, providing insights into the molecular mechanisms underlying semen quality changes. The study also established a cross-species human-bull sperm cell epigenetic clock, highlighting the potential for comparative studies on paternal biological aging.

**Conclusions:**

Our findings highlight the important role of DNA methylation regulation in sperm cell aging, providing a novel tool to evaluate reproductive potential in livestock. The creation of the bull sperm cell DNA methylation clock and the discovery of key molecular pathways linked to aging enhance our comprehension of sperm cell biology and provide insight into reproductive management and extending production longevity in cattle.

**Supplementary Information:**

The online version contains supplementary material available at 10.1186/s40104-026-01444-5.

## Background

Cattle are a major economic animal [1]. The development of artificial insemination technology has enabled an excellent bull to sire hundreds or thousands of offspring annually [2]. As germ cells, the sperm cells serve as the medium for transmitting the paternal genetic or epigenetic information to offspring, and they are directly linked to phenotypes such as reduced fertilization rates and higher risk of disease in offspring [3, 4]. Several studies have shown that the semen quality of bulls declines in older bulls [5].

The age of organisms is divided into chronological age and biological age. Chronological age is a direct measure of the elapsed time of an organism's life cycle. Biological age reflects how an organism's particular development is influenced by the passage of time. Generally, biological age is used to measure the degree of aging of the organism [6]. Biological age is measured by biomarkers that reflect the aging of biological functions, among which DNA methylation is considered the preferred biomarker [7, 8].

Researchers observed that age-associated changes in DNA methylation patterns, including site-specific hypo- and hypermethylation, are strongly related to organismal aging and can be leveraged to develop epigenetic clocks for predicting biological age [8–11]. Currently, various epigenetic clocks have been developed, such as ‘Horvath’ clock, ‘Hannum’ clock, ‘Pheno’ clock and ‘Grim’ clock [9, 12–14]. These epigenetic clocks are widely used to evaluate the health status of human body, explain the effects of various factors on lifespan, and aim to improve lifespan [15–17]. In addition, calculating epigenetic age acceleration (EAA) allows for the measurement of the gap between an individual's biological age and chronological age, which can be used to predict the individual's health status and provide insights for personalized health management [15–17]. Alterations in sperm DNA methylation levels and regional methylation patterns have been shown to be closely associated with fertility-related traits in bulls [18]. However, there is a lack of a sperm cell epigenetic clock that connects bull aging, DNA methylation, and bull fertility. Currently, research on the epigenetic clock of sperm cells is mainly focused on humans. The developed epigenetic clock can accurately evaluate the biological age of sperm cells and predict fertility through EAA [19, 20]. This can be used to predict the fertility rate of bulls [18, 21, 22].

The evidence highlights the importance of developing a DNA methylation clock for bull sperm cells and utilizing it to predict their reproductive potential. In this study, we generated DNA methylation profiles from bull sperm cells across a range of ages using reduced representation bisulfite sequencing (RRBS), and integrated these data with publicly available bisulfite sequencing datasets to develop and evaluate a sperm DNA methylation clock. This comprehensive dataset was used to develop a DNA methylation clock that accurately predicts the age of bulls. Furthermore, we investigated the association between EAA, semen quality records, and breeding values, all of which offer insights into the phenotype of the sires. This also reveals the potential molecular regulatory mechanisms of clock-related genes. The fundamental characteristics of sperm cell DNA methylation aging are shared between humans and cattle, and this study proposes an epigenetic clock applicable to both species. In summary, our developed clock serves as a predictor for the biological age of sperm cells from frozen semen and provides relevant indications for the productive lifespan. The human-cattle sperm cell DNA methylation clock reveals the potential to translate findings related to paternal aging between species.

## Methods

### Sample collection and DNA methylation data

In this study, commercial frozen semen samples were collected from healthy bulls and preserved and transported in liquid nitrogen. A total of 59 Holstein bulls were included, of which sperm genomic DNA was extracted for DNA methylation profiling. RRBS was performed for 49 bulls, and whole-genome bisulfite sequencing (WGBS) was conducted for 10 bulls. In addition to the in-house data, publicly available sperm DNA methylation datasets from Holstein bulls were integrated, including datasets from GSE131850, GSE119263, and GSE106538, as well as sperm DNA methylation data from Montbéliarde bulls deposited under PRJEB46371. Furthermore, human sperm DNA methylation data were obtained from GSE223748 and incorporated for cross-species analyses. The age and phenotype of all utilized samples are documented in Additional file 1: Table S1.

### RRBS library construction

Genomic DNA was extracted from bull semen samples using the TIANamp Genomic DNA Kit (TIANGEN, Beijing, China). DNA concentration was quantified using a Qubit fluorometer (Thermo Fisher Scientific), and DNA integrity was assessed by agarose gel electrophoresis. All DNA samples met the quality requirements for library construction. Lambda DNA was spiked into each sample to monitor bisulfite conversion efficiency. RRBS libraries were prepared following a standard protocol. In general, genomic DNA was digested with the methylation-insensitive restriction enzyme MspI, followed by end repair, dA-tailing, and ligation to Illumina sequencing adapters. DNA fragments were size-selected by gel excision to enrich fragments with insert sizes ranging from 40 to 220 bp, thereby enriching for CpG-dense regions. The size-selected DNA fragments were subjected to bisulfite conversion using the EZ DNA Methylation-Gold Kit (Zymo Research, Irvine, CA, USA), in which unmethylated cytosines are converted to uracils while methylated cytosines remain unchanged. Bisulfite-treated DNA was then PCR amplified to generate the final sequencing libraries. Library quality and fragment size distribution were assessed using an Agilent 2100 system, and library concentrations were determined by quantitative PCR. Paired-end sequencing was performed on NovaSeq 6000 platform according to the manufacturer’s instructions. RRBS libraries were prepared by Novogene Co., Ltd. (Beijing, China).

### WGBS library construction

Genomic DNA libraries were individually constructed for the 10 bulls. The qualified DNA was fragmented to 300 bp and subjected to end repair and A-tailing. Bisulfite conversion of DNA was performed using the ZYMO EZ DNA Methylation-Gold Kit. DNA fragments were amplified using PCR and size-selected. The quantified library was sequenced on the Illumina HiSeq X Ten platform (PE-150 bp FC; Novogene, Beijing, China).

### DNA methylation data analysis

WGBS raw reads were trimmed using Trim Galore v0.4.0 with default parameters. RRBS raw reads were trimmed using “--rrbs” parameters. The clean reads were mapped to the reference genome ARS-UCD1.2 using bismark v0.23.0 with default parameters and were deduplicated with the deduplicate_bismark options [23]. CpGs with a coverage of at least five and ten were used for further analysis. All downstream analyses were conducted using a minimum CpG coverage threshold of 5 reads, unless otherwise explicitly specified. The methylation levels of CpG sites were calculated using MethPipe v5.0.0, according to the number of methylated Cs in reads at the position corresponding to the site divided by the total of methylated Cs and unmethylated Ts mapping to that position [24]. Hypomethylated regions (HMRs) and differentially methylated regions (DMRs) were identified using the MethPipe software [24]. The mapping statistics are provided in Additional file 1: Table S2. HMRs and DMRs with ≤ 5 CpG sites were filtered out.

### Penalized regression models for epigenetic clock development

The development of epigenetic clocks for bulls began by retaining a set of consensus CpG sites that passed coverage filtering and were consistently detected across all samples within the dataset. An initial unsupervised hierarchical clustering analysis of methylation profiles was then performed to assess age-associated structure. Based on this analysis, a small number of samples showing extreme discordance between chronological age and methylation-based clustering patterns (for example, individuals aged ≥ 60 months clustering with the ≤ 25-month group) were excluded prior to downstream modeling, in order to avoid introducing pronounced cross–age-stage heterogeneity that could obscure the underlying age-associated signal. This filtering step was applied to preserve biological consistency rather than to optimize model performance. Subsequently, a unified workflow was applied to the resulting set of sufficiently abundant CpGs to identify clock CpG sites and develop the DNA methylation clocks (Additional file 2: Fig. S1). For individual CpGs, the associations between bull age and sperm cell methylation levels were assessed using CpGassoc [25]. Multiple-testing correction was applied using the Benjamini–Hochberg procedure, and CpG sites with FDR < 0.05 were defined as age-associated candidates. To further enrich biologically informative signals and avoid including CpGs with extremely limited dynamic range, the methylation standard deviation of each CpG was calculated across individuals, and only those within the upper quartile of the variability distribution were retained.

Subsequently, regression analyses were performed on the sets of consensus CpG sites obtained under different coverage depths, sequencing methods, and breeds to develop the corresponding sperm cell DNA methylation clocks under each condition, thereby developing distinct sperm cell epigenetic clocks for bulls. Penalized regression models were employed using the R function “glmnet” [26]. The α-value for the elastic net regression was fixed at 0.5, striking a balance between Ridge and Lasso type regressions, without optimizing for model performance. Leave-one-out cross-validation (LOOCV) was used to estimate the accuracy of the epigenetic clocks, whereby a single sample was iteratively excluded from the regression and its age was predicted. For each training set, optimal penalty parameters were automatically selected via tenfold internal cross-validation (cv.glmnet). Model performance was assessed using the Pearson correlation between predicted DNA methylation age and chronological age, together with the mean absolute error (MAE). Supplementary materials provide detailed information on the clock (CpG sites, genomic coordinates) and R software code.

### Relative age estimation

To introduce biological significance into age estimation for species with substantial differences in lifespan, such as cattle and humans, and to address the unavoidable bias caused by the uneven distribution of data points across various age ranges for these species, the following formula was employed for estimating relative age: Relative Age = Age/Maximum Lifespan. The maximum lifespan for both species was sourced from the AnAge database [27].

### Moderation analysis

To ascertain the factors influencing the relationship between chronological age and predicted age, we employed linear models and calculated *P* for each term using linear regression and the “lm” function in R. A diverse range of bulls covering various phenotypes was selected for training the model. The moderating factors we tested were semen quality and the breeding values of the bulls. By inputting a matrix of factors as independent variables and the epigenetic age prediction as the dependent variable, we assessed the significance of each factor.

### Epigenetic pacemaker

The Python package EpigeneticPacemaker was used to generate predictions for each bull’s epigenetic state [28].

### Genome-wide association study (GWAS) enrichment analysis

The significant SNPs for all analyzed traits of cattle were obtained via public GWAS (*n* = 27,143; https://figshare.com/s/ea726fa95a5bac158ac1) [29–31]. Finally, SNPs and different clock-associated genomic regions were combined for integrated analysis to determine the degree of correlation between these regions and 49 complex traits.

### Cross-species and cross-breed datasets for sperm DNA methylation clock development

Publicly available human and bovine sperm DNA methylation datasets were incorporated to support cross-species and cross-breed analyses. Human sperm DNA methylation data were obtained from GEO GSE223748, comprising 379 samples with recorded chronological ages (in years).

Sperm DNA methylation data from Montbéliarde bulls were retrieved from BioProject PRJEB46371. RRBS samples lacking age information were excluded based on metadata curation. After filtering, 61 Montbéliarde bulls with ages ranging from 15–39 months were retained. All samples were processed using the same RRBS analysis pipeline as applied to the in-house bull sperm data and were subsequently included in downstream epigenetic clock analyses.

### Statistics and reproducibility

The sample size (*n*, representing biological replicates) is indicated in the figure legends and Supplementary Tables. Statistical analyses were performed in R (v4.0.3) using the packages CpGassoc and glmnet (v4.1.4). Comparisons of DNA methylation levels among different age stages were conducted using Wilcoxon rank-sum tests. Associations between chronological age and CpG methylation levels were assessed by linear regression implemented in CpGassoc. Epigenetic clocks were developed using elastic net regression (*α* = 0.5) with leave-one-out cross-validation, and accuracy was evaluated by the Pearson correlation coefficient (*r*) and MAE. Moderation analyses were carried out using multiple linear regression in R. All tests were two-sided, and statistical significance was defined as *P* < 0.05 (*).

## Results

### The distribution of DNA methylation levels across different age stages

To investigate the distribution of DNA methylation levels in bull sperm cells across different age stages, we performed unsupervised hierarchical clustering analysis based on RRBS-derived DNA methylation profiles. The analysis was conducted using consensus CpG sites covered across all samples, with a minimum sequencing coverage of 10 × to ensure reliable methylation quantification, in conjunction with chronological age. RRBS-based DNA methylation profiling was performed on semen samples from 49 bulls aged 21–115 months (Additional file 2: Fig. S2A; Additional file 1: Table S1). This analysis revealed three major age-associated clusters, corresponding to bulls aged “ ≤ 25 months”, “26–59 months”, and “ ≥ 60 months”. Notably, a subset of individuals did not conform to the primary age-stage clustering pattern. These outlier samples were excluded from subsequent analyses. After this filtering step, a total of 35 individuals were retained for downstream analyses, which enabled a clearer characterization of methylation dynamics across distinct age stages (Additional file 2: Fig. S2B). In this part of our study, an increase in the overall DNA methylation levels was observed in bull sperm cells as age progressed in months (Additional file 2: Fig. S2C), consistent with previous observations in humans [32].

**Fig. 1:**
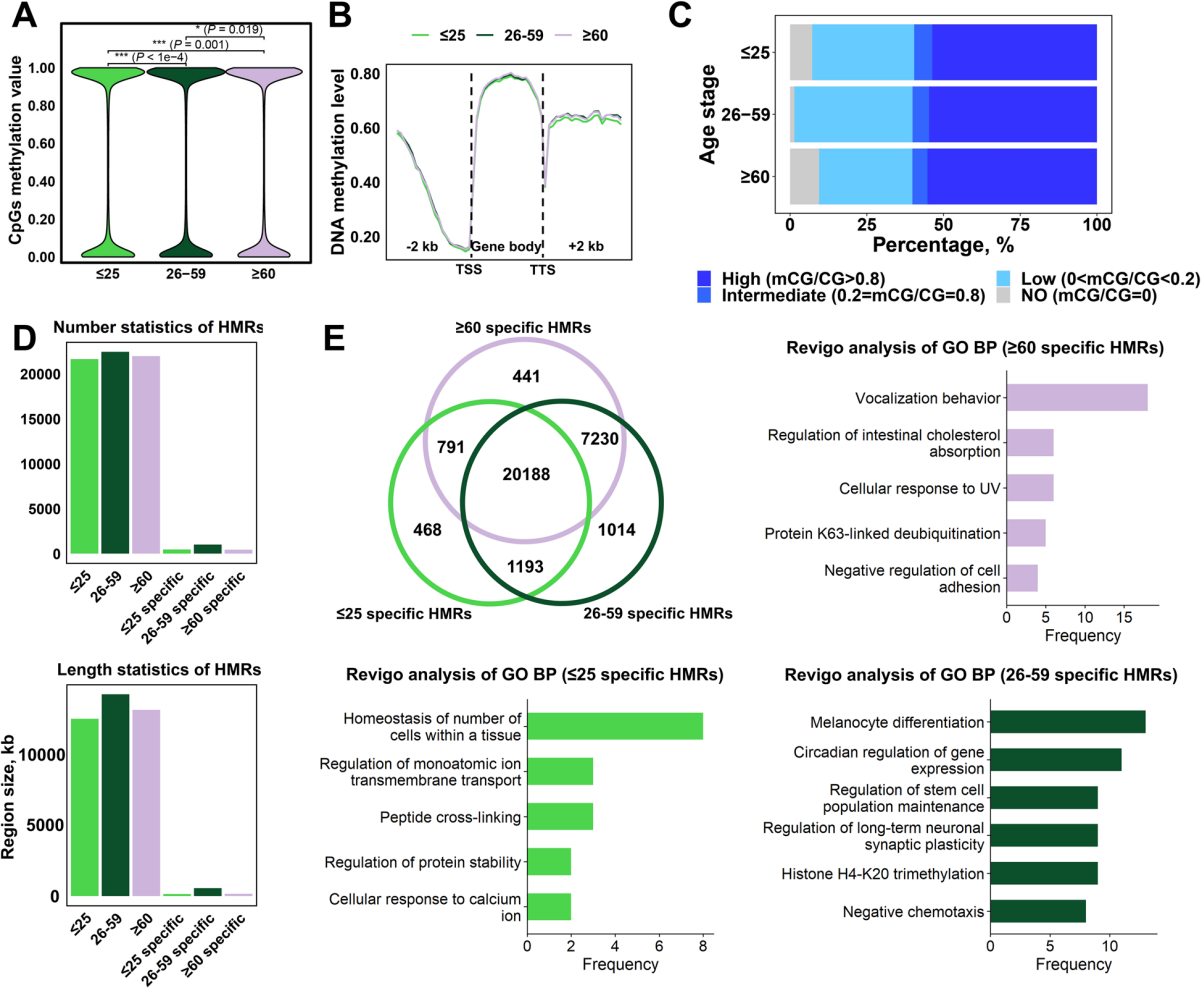
Displays the distribution of DNA methylation levels in bull sperm cells across different age stages. **A** Wilcoxon rank-sum test for per-sample average methylation levels across all CpG sites among different age stages. **B** The average DNA methylation levels in the promoter regions (− 2 kb), gene bodies, and downstream regions (+ 2 kb) across bull sperm cell samples from different age stages. The gene body, promoter, and downstream regions of genes are divided into 20 equally sized bins. **C** The proportional distribution of CpG methylation states across age stages, with the average methylation level for each bin calculated as the ratio of methylated cytosine to total cytosine sequenced. **D** Upper panel shows the number of hypomethylated regions (HMRs) across different age stages, including both total and age-stage-specific (unique) HMRs. Lower panel shows the total genomic length of HMRs corresponding to each category. Age-stage-specific HMRs refer to hypomethylated regions uniquely identified in a given age stage and absent from the other two stages. **E** A Venn diagram shows the overlap and age-stage-specific HMRs identified across different age stages. Bar charts, color-coded to match the Venn diagram, present Gene Ontology (GO) Biological Process (BP) enrichment results summarized by Revigo for genes associated with HMRs specific to each age stage

The average methylation level across all CpG sites was calculated for each sample, and pairwise Wilcoxon rank-sum tests were performed between age stages. Highly significant differences were observed among the stages (Fig. 1A). To characterize the dynamics of DNA methylation across the bull sperm cell genome at various age stages, the average methylation levels were measured within gene bodies and their corresponding upstream and downstream 2 kb regions. CpG methylation levels were observed to decrease within gene promoters, reaching their lowest point at the transcription start site (TSS), and then exhibiting a continuous increase from the TSS to the transcription termination site (TTS), with the “ ≤ 25 months” age stage showing the lowest levels downstream of the TTS (Fig. 1B). We subsequently focused on examining the proportion of different methylation levels across different age stages. It was observed that the “26–59 months” stage showed the lowest proportion of CpG sites in the unmethylated category (mCG/CG = 0) compared with the other age stages (Fig. 1C). Further analyses of the number and genomic length of HMRs across different age stages revealed that both the total number and total genomic length of HMRs were highest in the 26–59 months stage, followed by the ≥ 60 months and ≤ 25 months stages. Notably, age-stage-specific (unique) HMRs, defined as regions present exclusively in a single age stage, were observed in all three age stages, as evidenced by their numbers and total genomic lengths. Given that hypomethylated regions are commonly associated with transcriptionally permissive chromatin states, these age-stage-specific HMRs may be linked to stage-associated regulatory activity (Fig. 1D).

Moreover, age-stage-specific HMRs were further characterized. Gene Ontology (GO) enrichment analysis revealed that age-stage-specific HMRs in the “ ≤ 25 months” stage were predominantly associated with biological processes related to cellular function and homeostasis, including homeostasis of cell number within a tissue and regulation of monoatomic ion transmembrane transport. In the “26–59 months” stage, age-stage-specific HMRs were enriched in processes related to cellular development and regulation of gene expression, such as melanocyte differentiation and circadian regulation of gene expression. In the “ ≥ 60 months” stage, age-stage-specific HMRs were enriched in a set of broader biological process terms, including vocalization behavior, regulation of intestinal cholesterol absorption, and cellular response to ultraviolet radiation (Fig. 1E). We note that some of these GO terms are not directly specific to sperm biology and may reflect broader functional annotations of genes with pleiotropic regulatory roles.

### Identification of age-associated CpG features for sperm cell DNA methylation clock development in stud bulls

**Fig. 2:**
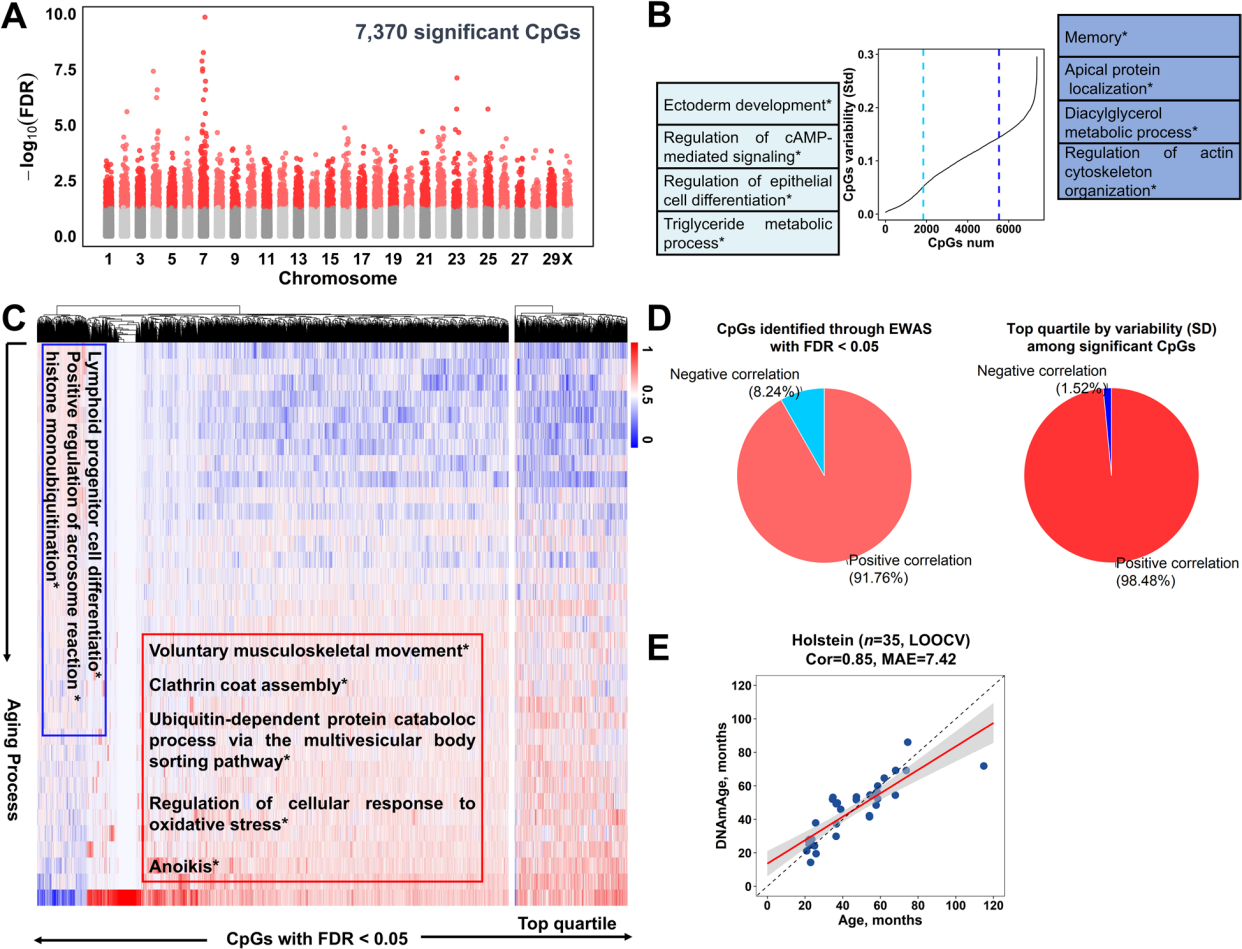
Details the process of developing an epigenetic clock based on sperm cell samples from each age stage. **A** A small-scale epigenome-wide association study (EWAS) was conducted to screen for age-associated CpGs. **B** The left and right panels display functional annotations of genes associated with CpGs in the lower and upper quartiles of standard deviation, respectively, using Revigo to annotate Gene Ontology (GO) Biological Process (BP) terms. **C** A heatmap shows age-related methylation patterns of EWAS-significant CpGs (FDR < 0.05) with samples ordered by chronological age. The right panel highlights the subset of significant CpGs in the upper quartile of methylation standard deviation. **D** Pie charts represent the percentage of age-characteristic CpGs with positive and negative correlations with age. The left and right charts display EWAS-significant CpG sites (FDR < 0.05) and CpG sites in the upper quartile of standard deviation, respectively. **E** A preliminary epigenetic clock for bull sperm cells was developed using 35 RRBS samples. The sample size (*n*), correlation coefficient (Cor), and mean absolute error (MAE) are all reported

To prioritize age-associated CpG features for epigenetic clock development, we implemented an integrated analytical framework to systematically select informative CpG sites and build the epigenetic clock (Additional file 2: Fig. S1). Within this framework, we first performed a small-scale epigenome-wide association study (EWAS) on 35 sperm cell samples from Holstein bulls that conformed to the unsupervised age-stage clustering, leading to the identification of 7,370 age-associated CpG sites (FDR < 0.05, Fig. 2A). Based on the standard deviation, we measured the variability of these CpGs and selected those in the upper quartile of variability for further analysis. Genes associated with these CpGs were enriched in pathways related to signal transduction and protein localization, such as Memory and Apical protein localization (Fig. 2B). To further characterize the age-related methylation dynamics of these CpGs, we visualized the age-dependent methylation changes of the FDR-significant CpG sites using a heatmap, with samples ordered by chronological age (Fig. 2C). The right panel of the heatmap highlights the subset of FDR-significant CpGs in the upper quartile of variability, illustrating their methylation trajectories across age. We next quantified the directionality of age-associated methylation changes. Among all FDR-significant CpGs, the majority showed increasing methylation with age, accounting for 91.76% of sites (Fig. 2D, left). This trend was further accentuated in the high-variability subset, where 98.48% of CpGs exhibited age-increasing methylation (Fig. 2D, right). Using these highly variable, age-related CpGs derived from the age-stage samples (*n* = 35), a preliminary epigenetic clock for bull sperm cells was developed (Additional file 1: Table S3). We used cross-validation to obtain unbiased estimates of the age correlation coefficient *r*, defined as the Pearson correlation between estimated DNAmAge and known chronological age, and the mean absolute error (MAE; in units of months). For the bull clock, we observed high cross-validation estimates of the correlation (*r* = 0.85), with a corresponding MAE of 7.42 (Fig. 2E).

### An integrated DNA methylation clock for Holstein bulls using in-house and public datasets

**Fig. 3:**
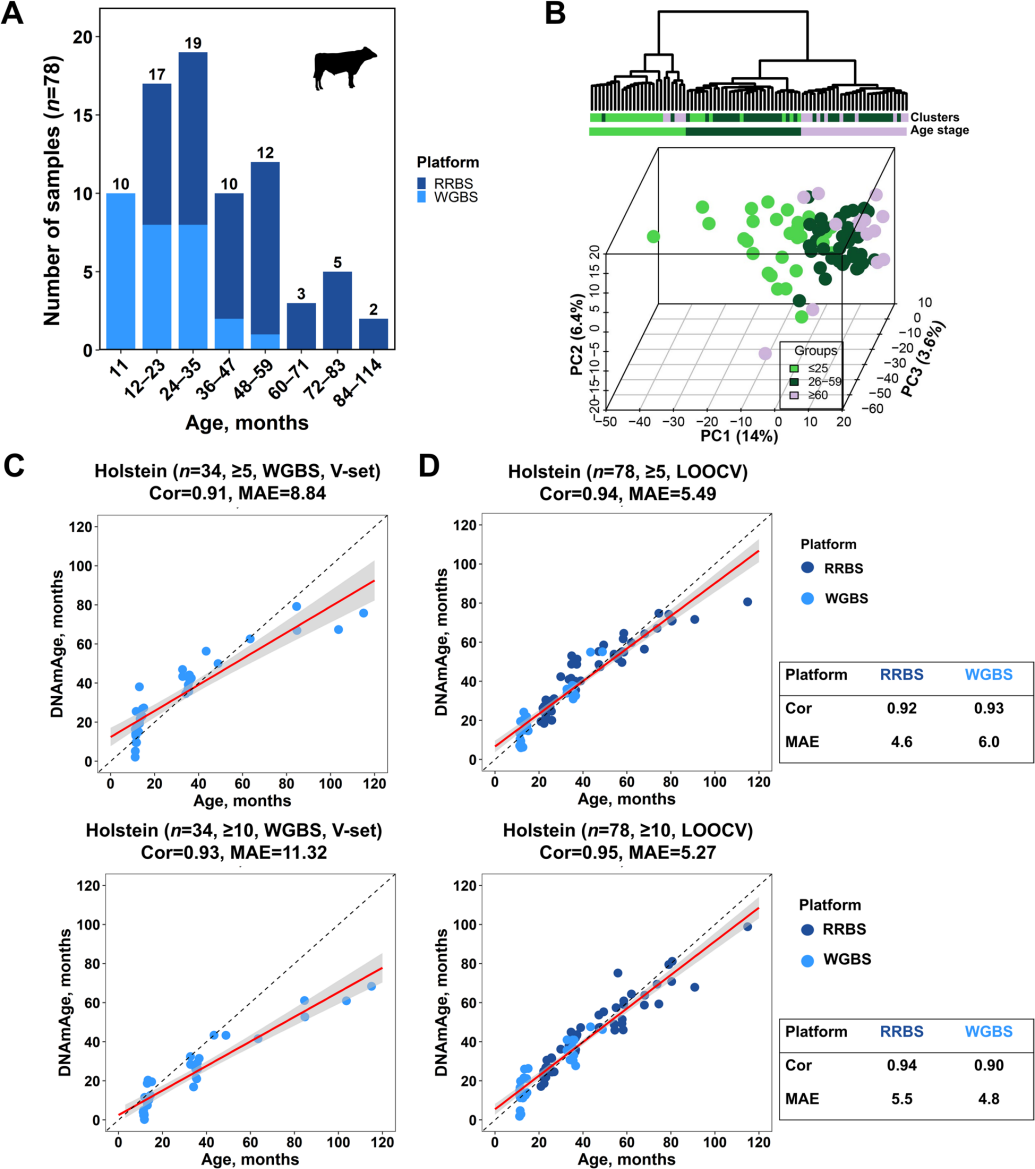
Integration of whole-genome bisulfite sequencing (WGBS) and reduced-representation bisulfite sequencing (RRBS) data for epigenetic clock development. **A** Bar chart showing the distribution of bull sperm cell samples across different age bins after integrating WGBS data, with dark blue representing RRBS samples and light blue representing WGBS samples. **B** Unsupervised hierarchical clustering and three-dimensional principal component analysis (PCA) of bull sperm cell DNA methylation profiles after integration of WGBS and RRBS data. **C** Transferability assessment: development of bull sperm cell DNA methylation clocks using RRBS samples under different sequencing coverage thresholds (≥ 5 or ≥ 10), with validation performed on independent WGBS samples. **D** Development of cross-platform bull sperm cell DNA methylation clocks using combined RRBS and WGBS data under different sequencing coverage thresholds (≥ 5 or ≥ 10). Correlation coefficients (Cor), mean absolute error (MAE), and sample sizes (*n*) for RRBS and WGBS samples are reported. Dark blue points represent RRBS data, and light blue points represent WGBS data. The table on the right summarizes Cor and MAE values stratified by sequencing platform

To develop a more universally applicable bull sperm cell DNA methylation clock and to assess its stability, additional WGBS data from known-aged Holstein bull sperm cells were incorporated, including 10 WGBS samples generated and 24 publicly available WGBS samples, totaling 34 individuals. These WGBS data spanned an age range of 12–116 months and were used to complement the age distribution of the RRBS-based dataset (Fig. 3A and Additional file 1: Table S1). We then performed an unsupervised hierarchical clustering analysis across all samples. Consistent age-stage clustering patterns were observed in the integrated dataset, recapitulating the same three age-stage clusters identified in the unsupervised analysis of the 49 RRBS samples, while the deviation pattern was not entirely identical to that observed in the RRBS analysis, with only a few individuals showing clear and extreme age-stage discordance relative to their chronological age (e.g., samples ≥ 60 months clustering with the ≤ 25-month group). Given the overall reproducibility of these clustering patterns across datasets, we excluded these markedly discordant individuals during the development of the Holstein bull sperm epigenetic clock, retaining 78 individuals for subsequent analyses (Fig. 3B; Additional file 2: Fig. S3; Additional file 1: Table S1). To evaluate the feasibility of cross-platform integration for DNA methylation clock development, we applied the RRBS-derived clock to WGBS data. Under commonly adopted CpG coverage thresholds of 5 × and 10 ×, the clocks showed high transferability, with correlation coefficients of 0.91 and 0.93 and MAEs of 8.84 and 11.32 months, respectively (Fig. 3C; Additional file 2: Fig. S4A and S4B; Additional file 1: Table S4).

Following the workflow described in the Methods, we developed the DNA methylation clock using data from 78 bulls. In parallel, we evaluated clock performance under two commonly used CpG retention criteria based on minimum coverage thresholds of 5 × and 10 ×. Notably, whether a read coverage threshold of ≥ 5 or ≥ 10 was chosen, clocks with high correlation coefficients were obtained. When the read coverage was ≥ 5, the correlation coefficient between chronological age and predicted age was 0.94, with a MAE of 5.49 months. When the read coverage was increased to ≥ 10, the correlation coefficient was 0.95, with a MAE of 5.27 months, indicating a superior model performance at the higher sequencing coverage (Fig. 3D, Additional file 1: Table S4). External validation of the integrated clock, developed using data from 78 bulls with a minimum coverage of 10 ×, in an independent Holstein WGBS cohort (*n* = 9) also showed overall concordance between chronological age and predicted age (Cor = 0.88, MAE = 7.08 months), although predictions for relatively older individuals should still be interpreted more cautiously (Additional file 2: Fig. S4C).

By integrating all clock models and examining the intersection of clock CpG sites derived under different sample size and sequencing coverage criteria, we observed a limited overlap of CpG sites across standards. Specifically, the clock developed from RRBS data with read coverage ≥ 5 and the clock based on RRBS data with read coverage ≥ 10 shared only 13 overlapping clock sites (Additional file 2: Fig. S5).

### Moderation analysis

To identify factors influencing the relationship between chronological age and predicted age, we employed multiple linear regression using epigenetic age acceleration, defined as the residuals from regressing predicted age on chronological age, and calculated the *P*-value for each term to assess associations with age acceleration. Analyses were based on the optimal DNA methylation clock developed from 78 bulls using the 10 × coverage threshold. Within the Holstein bull dataset, various factors underwent significant moderation testing, specifically semen quality traits and breeding value traits. For each factor, we assessed its significance by fitting a moderation model that included chronological age, the target factor, and their interaction term as predictors of predicted age. We found that among all the factors tested, fresh semen motility at first ejaculation, frozen semen abnormality rate, and testicular circumference were three significant moderating factors (Table 1, Additional file 1: Table S5 and S6). This indicates that these three traits were significantly associated with epigenetic aging in bulls.

**Table 1:** Multivariate linear regression to explore the association between epigenetic acceleration and different traits

Trait	*P* for trait	*P* for age	*P* for interaction
MS_1st	0.0031	0.0095	0.0021
Testicular circumference	0.0054	0.0001	0.0032
ARAF	0.0232	0.0033	0.0218
NMSPE_1st	0.0584	0.4349	0.0668
Rate of abnormality	0.1142	0.0332	0.1101

The significance of each factor was assessed based on the *P* associated with the factor and interaction terms in each model. MS_1st: sperm motility at first ejaculation. ARAF: abnormal rate after freezing. NMSPE_1st: number of total motile sperm at first ejaculation.

### Functional annotation of regions associated with epigenetic acceleration

**Fig. 4:**
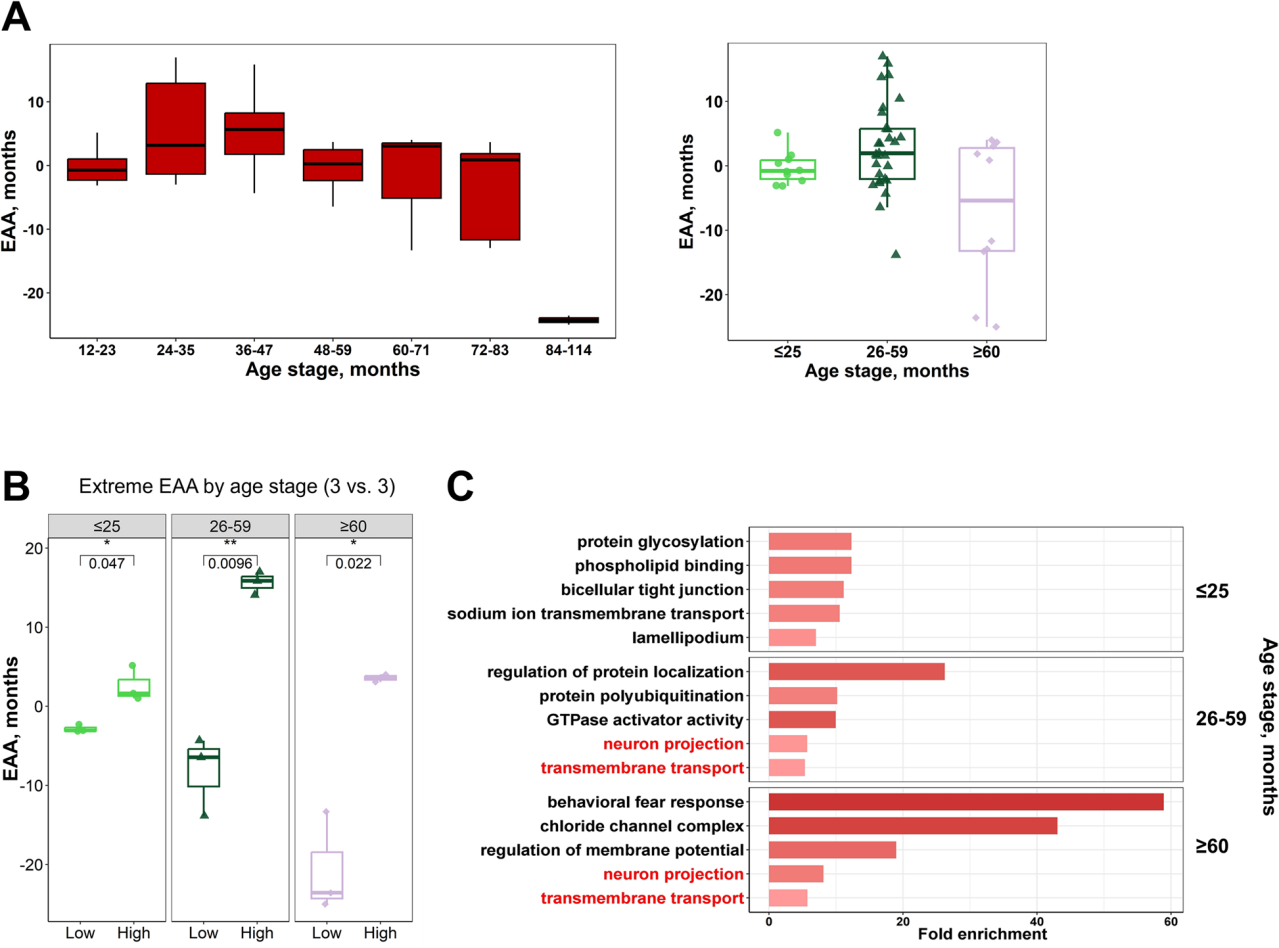
Annotations of genomic regions associated with epigenetic acceleration based on reduced-representation bisulfite sequencing (RRBS) platform data. **A** Box plots showing epigenetic age acceleration (EAA), defined as the residuals from the regression of DNAmAge on chronological age, across different age bins and age stages in bull sperm samples profiled by RRBS. **B** Within each age stage (≤ 25 months, 26–59 months, and ≥ 60 months), the three individuals with the highest and lowest EAA values were selected. Box plots display the distribution of EAA for these extreme individuals within each age stage, with statistical significance assessed by pairwise comparisons. **C** Functional annotation of differentially methylated regions (DMRs) associated with epigenetic acceleration within each age stage. Gene Ontology (GO) Biological Process (BP) enrichment results were summarized using Revigo, highlighting age-stage-specific functional categories associated with EAA

To investigate how epigenetic age acceleration influences semen quality traits, we first examined the distribution of epigenetic acceleration across different year- and age-stage groups. We observed that epigenetic acceleration exhibited a non-linear trend with increasing age stage, showing an initial increase followed by a decrease, suggesting that bull sperm cells dynamically age or rejuvenate at the DNA methylation level relative to their chronological age across physiological stages. Epigenetic acceleration in the age stages of ≤ 25 months and ≥ 60 months showed a median less than 0, indicating epigenetic rejuvenation, while in the 26–59 months age stage, epigenetic acceleration exhibited a median greater than 0 (Fig. 4A).

Furthermore, based on the age-stage stratification established in Fig. 2, we selected three individuals with the highest and three with the lowest epigenetic acceleration within each age stage and performed pairwise differential DNA methylation analyses (3 vs. 3) to investigate molecular features associated with accelerated versus decelerated epigenetic aging (Fig. 4B). GO BP term Revigo analysis of genes overlapping with differential DNA methylation regions related to epigenetic acceleration across different age stages revealed neuron projection and transmembrane transport as common pathways associated with epigenetic acceleration in the 26–59 months and ≥ 60 months age stages. While neuron projection may have indirect roles through broader cellular regulatory mechanisms, transmembrane transport is more directly related to the regulation of spermatogenesis and cellular function (Fig. 4C).

### GWAS enrichment analysis for sperm cell DNA methylation clock

**Fig. 5:**
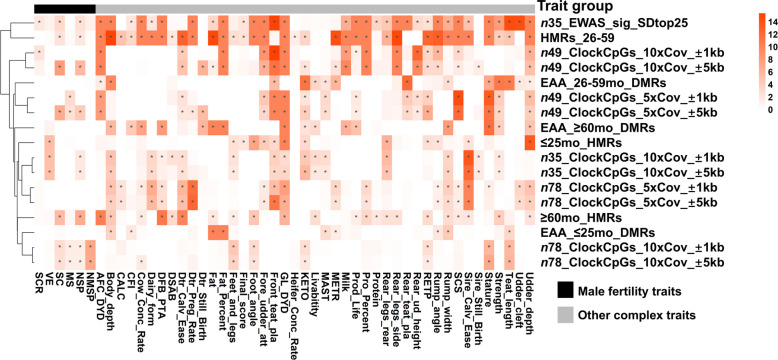
GWAS enrichment analysis of 49 cattle traits. VE: volume of the ejaculate. SC: the sperm concentration per ejaculate. MS: the initial sperm motility. NSP: the number of sperm per ejaculate. NMSP: the number of motile sperm per ejaculate. SCR: sire conception rate. ^*^P < 0.05

To assess and annotate regions associated with the sperm methylation clock, we conducted GWAS signal enrichment analysis on DNA methylation regions linked to different clock features. This analysis revealed common GWAS signal enrichments, including 49 cattle traits such as semen quality traits (VE, SC, MS, NSP, and NMSP) and sire conception rate (SCR), across genomic regions of different clock features. As shown in Fig. 5, sperm cell DNA methylation clock regions, whether under different sequencing platforms, sample sizes, or filter criteria, as well as larger genomic age-related methylation regions, exhibited enrichment of GWAS signals for various cattle complex traits. This suggests that clock-associated regions may contribute to the regulation of complex traits and the aging process to varying extents. Notably, semen quality traits and SCR were both significantly enriched, with regions showing significant enrichment of semen quality trait GWAS signals being more numerous than those for SCR.

### Exploring the potential of sperm epigenetic clocks across species, breeds, and age stages

Age-associated DNA methylation changes are typically non-linear over time, with faster rates of change in early life that decrease as age advances. To test whether this is occurring in our samples, we employed the epigenetic pacemaker model, an approach developed in previous studies. This approach is complementary to the regression-based approach used in epigenetic clocks. Previous studies have shown that in humans the logarithmic function provides a good fit for the association of epigenetic age with chronological age. The same was also shown in dogs [33]. In our bull samples, we found that the relationship between epigenetic state and chronological age is well fit by a square root function (Additional file 2: Fig. S6). These results are consistent with those observed in humans and dogs, indicating that the rate of DNA methylation changes decreases with increasing age of bulls.

**Fig. 6:**
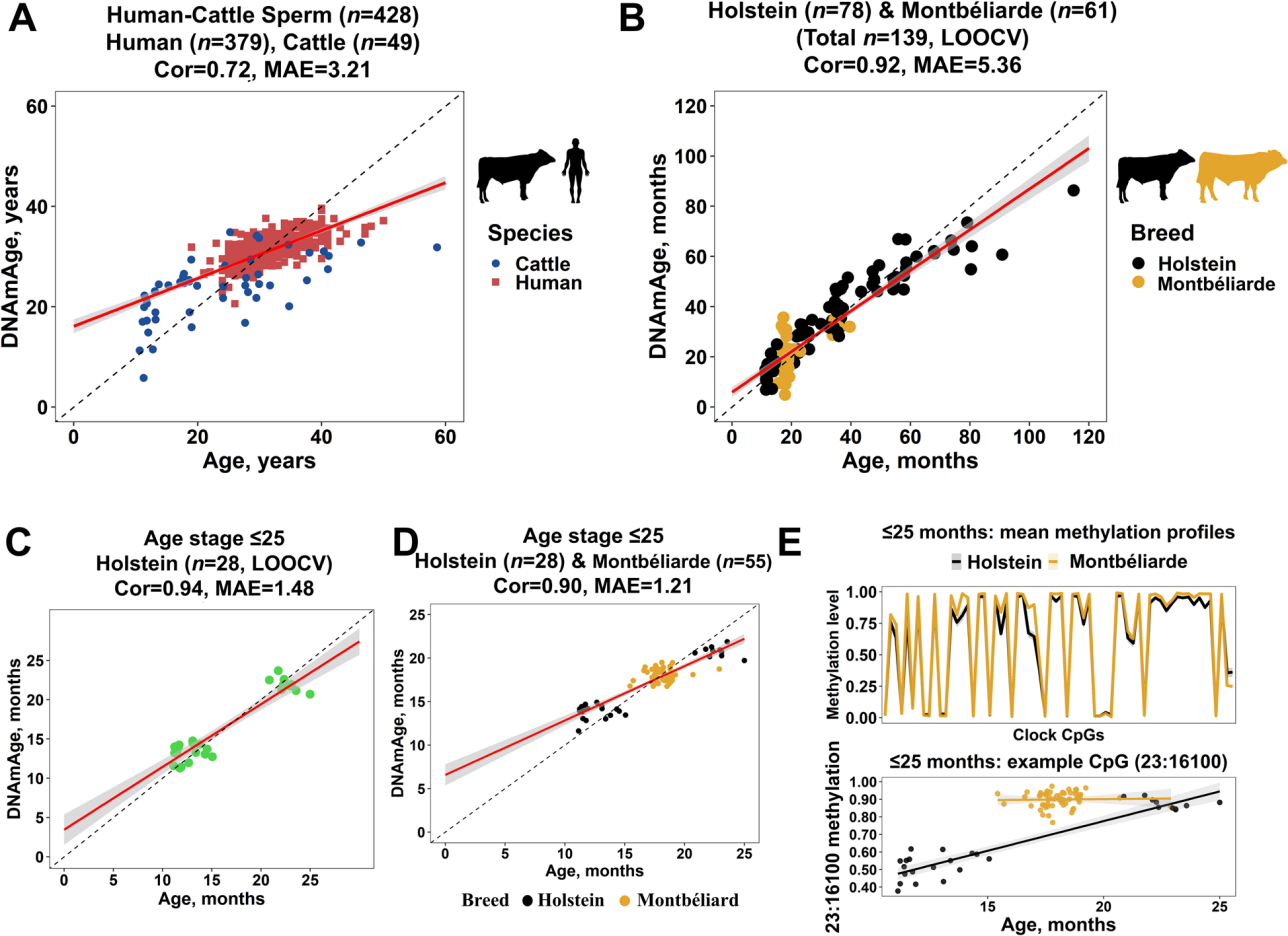
Exploring the potential of sperm epigenetic clocks across species, breeds, and age stages. **A** Development of a cross-species sperm epigenetic clock integrating human and cattle sperm DNA methylation data. The model was trained on combined datasets (Human, *n* = 379; Cattle, *n* = 49), and the correlation (Cor) and mean absolute error (MAE) are reported. **B** Development of a cross-breed sperm epigenetic clock using bull sperm samples from Holstein (*n* = 78) and Montbéliarde (*n* = 61) breeds, evaluated by leave-one-out cross-validation (LOOCV). **C** Performance of an age-stage-specific sperm epigenetic clock for young bulls (≤ 25 months) developed using Holstein samples (*n* = 28) and assessed by LOOCV. **D** Cross-breed evaluation of the ≤ 25-month age-stage-specific sperm epigenetic clock integrating Holstein (*n* = 28) and Montbéliarde (*n* = 55) samples, illustrating the consistency of age prediction across breeds within the same age stage. **E** Age-stage-specific methylation patterns of clock CpG sites in young bulls (≤ 25 months). The upper panel shows the mean methylation profiles of clock CpGs for Holstein and Montbéliarde breeds, while the lower panel presents an example clock CpG site (Chr23:16100) demonstrating breed-specific methylation dynamics with age

To explore the potential of cross-species sperm epigenetic clocks, we performed reciprocal age-scale transformations between humans and cattle and developed corresponding epigenetic clock models. Specifically, bovine chronological age (in months) was converted to human chronological age (in years), and conversely, human age was converted to the bovine age scale, using a simple age transformation scheme. These transformed age variables were then used as response variables to develop sperm DNA methylation clocks based on the integrated human and cattle datasets. Using this approach, the resulting cross-species clock captured a general concordance between predicted DNA methylation age and the transformed chronological age across species (Fig. 6A; Additional file 2: Fig. S7A; Additional file 1: Table S7). Notably, the transformation of maximum age was treated conservatively and was intended solely to align the overall age ranges between species, rather than to imply precise equivalence of lifespan or aging dynamics. Together, these results suggest that, under simple age-scale transformations, sperm DNA methylation patterns exhibit shared age-associated signals across species.

As shown in Fig. 6B, a combined cattle sperm DNA methylation clock was developed using Holstein (*n* = 78) and Montbéliarde (*n* = 61) samples and evaluated by LOOCV. The model exhibited a strong correlation between chronological age and predicted DNAm age (Cor = 0.92), with a MAE of 5.36 months, indicating robust predictive performance across breeds. Given the pronounced clustering of younger individuals, we further developed age-stage-specific clocks. For bulls aged ≤ 25 months, a Holstein-specific sperm DNA methylation clock achieved a very high prediction accuracy under LOOCV (Fig. 6C), with a correlation of 0.94 and an MAE of 1.48 months. Notably, similar clock development was also attempted for the other two age stages (26–59 months and ≥ 60 months). While the intermediate age stage (26–59 months) yielded a moderate predictive performance (Additional file 2: Fig. S7B and S7C), the ≥ 60-month stage did not produce a clock with comparably high correlation, likely due to the limited sample size in this age group. Consistently, stage-specific evaluation of a unified clock further revealed increased prediction error and systematic underestimation in bulls aged ≥ 60 months (Additional file 2: Fig. S7D).

To assess whether age-related methylation signatures were conserved across breeds at early life stages, a combined Holstein (*n* = 28) and Montbéliarde (*n* = 55) clock was developed for bulls aged ≤ 25 months (Fig. 6D, Additional file 1: Table S8) [18]. This model maintained strong predictive accuracy (Cor = 0.90, MAE = 1.21 months), supporting the presence of shared epigenetic aging patterns in young bulls across breeds. As shown in Fig. 6E (upper panel), the mean methylation profiles of clock CpGs at the ≤ 25-month stage were highly similar between Holstein and Montbéliarde bulls, indicating broadly conserved age-associated methylation patterns across breeds. In contrast, the lower panel of Fig. 6E highlights a representative CpG site exhibiting divergent methylation trajectories between the two breeds, illustrating that despite overall concordance at the global level, breed-specific variability persists at individual CpG loci.

## Discussion

Epigenetic aging clocks, originally developed for humans, have been widely used in medical research and clinical trials to assess biological aging and even predict mortality risk [9, 12, 34–36]. Although epigenetic aging clocks have expanded to various species, their application in male animal sperm is limited and mainly focused on humans [3, 20, 36, 37]. In this study, we found that 91.76% of age-associated CpG sites exhibited a positive correlation with age, which is consistent with previous studies [38, 39]. By integrating WGBS data, we developed, to our knowledge, the first epigenetic clock that accurately predicts bull sperm age across sequencing platforms (*r* = 0.95, MAE = 5.27), with external validation in an independent Holstein cohort confirming these results. EAA-associated genes are enriched in transmembrane transport pathways. It is believed that the genetic architecture underlying age-related traits is somewhat conserved between humans and animals [36, 40–42]. Our research, for the first time, demonstrates the existence of orthologous epigenetic clock CpG sites between bull and human sperm cells, identified through genomic liftover, enabling cross-species age prediction (*r* = 0.72, MAE = 3.21). In summary, our results fill gaps in data and theory regarding sperm cell epigenetic aging in the field of aging research. Because the overall sample size, particularly the number of bulls in the relatively older age group (≥ 60 months), was limited in the current dataset, the robustness and generalizability of the model and the interpretation of age-related methylation dynamics should be approached with caution. Additionally, although our integrative analyses provide biologically relevant support for the identified age- and clock-related signals, direct functional validation of the implicated genes and pathways remains to be established in future studies.

We have demonstrated that stage-specific DNA methylation profiles better reflect the characteristics of sperm cells at different age stages compared to traditional growth and developmental classifications. Hypomethylated regions (HMRs) play a crucial role in gene regulation and are essential for maintaining sperm function [43]. In the three methylome-defined age stages, stage-specific HMRs showed distinct but broadly interpretable enrichment patterns. In bulls aged ≤ 25 months, enriched terms were mainly related to cellular homeostasis and ion transport, consistent with general processes involved in spermatogenesis and protein turnover [44, 45]. In the 26–59 months stage, enrichment was observed for gene regulatory processes, including circadian regulation and stem cell–related terms, which may reflect broader regulatory features associated with reproductive physiology [46, 47]. In the relatively older age group (≥ 60 months), HMRs were enriched in a set of more heterogeneous GO terms, including cholesterol absorption, vocalization behavior, and cellular response to ultraviolet radiation. We note that some of these terms are not directly specific to sperm biology and may arise from broader functional annotations of pleiotropic or relatively long genes, as well as inherent biases in GO enrichment analyses. Nonetheless, certain pathways, such as those related to lipid metabolism (e.g., cholesterol), may still have indirect relevance to reproductive function through their roles in steroid hormone synthesis [48], whereas environmental response–related terms (e.g., UV response) may reflect general genomic stress or damage-associated processes [49].

Age-related and complex trait-associated changes in DNA methylation often follow spatial correlation patterns [50, 51]. Notably, the RRBS-based clock (≥ 5 reads) demonstrated the highest accuracy (*r* = 0.96). Several studies have explored the development of epigenetic clocks in cattle. An oocyte clock developed using the HorvathMammalian40K array achieved high accuracy (*r* = 0.919) [52]. A clock for tropically adapted cattle, developed from tail hair and portable sequencing devices, also showed robust performance [53]. Another clock using TSU samples with the HorvathMammalMethyl40K array demonstrated high accuracy (*r* > 0.97) with just 217 CpG sites [54]. A blood-based clock, developed from 96 Holstein cows using targeted bisulfite sequencing, achieved a correlation of 0.88 and a MAE of 9.35 [55]. Notably, the methylation patterns and evolutionary rates of sperm differ significantly from those of somatic tissues, a phenomenon also observed in primates [56]. The linear predictive model of the bovine sperm cell clock reflects the shared aging accumulation between sperm cells and somatic cells. Our cross-breed clock samples were primarily concentrated in the ≤ 25 months stage, revealing shared methylation patterns between Montbéliarde and Holstein bulls during this early developmental phase. Interestingly, we found that the methylation levels of CpG sites associated with the cross-breed clock exhibited divergent age-related trends across breeds, suggesting that breed-specific factors may influence aging patterns of DNA methylation [42]. Although previous studies in dogs found no significant correlation between EAA and breed-specific traits such as average lifespan, maximum median lifespan, average weight, and average height, the varying EAA levels observed across different breeds and age stages may reflect breed-specific phenotypic developmental processes.

Recent studies have found no significant correlation between semen parameters and EAA in human sperm but did observe associations with abnormalities in sperm head morphology—parameters typically not measured in routine semen analysis [37]. Similar experimental designs have shown that advanced sperm epigenetic age is associated with prolonged time-to-pregnancy for couples trying to conceive and a shorter gestation period for their offspring, independent of maternal contributions [20]. However, as some samples in the study were collected overnight without assessing fresh sperm motility. In cattle, a uniform rearing environment combined with detailed pedigree and semen quality records makes sperm an excellent model for studying the relationship between physiological aging and complex traits [57, 58]. In our study, we found that semen quality traits, such as sperm motility and abnormal rate, were significantly associated with EAA. Sawant et al. [37] reported a negative correlation between EAA and sperm head elongation factor, indicating that higher epigenetic age is associated with reduced sperm head elongation. This reduction results in a blunter and shorter sperm head, impairing the sperm's ability to swim efficiently [59]. Reduced motility decreases the likelihood of sperm successfully reaching and fertilizing the oocyte, leading to prolonged time-to-pregnancy. This finding is consistent with results observed in humans. It cannot be guaranteed that interventions aimed at reversing epigenetic age in animal models will translate successfully to humans. However, using appropriate animal models such as bulls to investigate the relationship between sperm aging and semen quality significantly increases the likelihood of success. Folic acid and vitamin B12 supplementation altered sperm DNA methylation in a region-specific manner, suggesting that dietary intervention may provide a useful approach to investigate modulation of sperm DNA age and its effects on semen quality [60].

Understanding the conservation of sperm aging mechanisms between humans and cattle could not only establish cattle as a potential model for studying human reproductive diseases but also enhance cattle genetic improvement programs by leveraging insights from human research. Using homologous CpG sites, we developed a cross-species sperm cell epigenetic clock for humans and cattle, highlighting conserved methylation patterns and the shared impact of aging. Human-specific hypomethylated promoters are linked to neural development, while cattle-specific promoters are associated with lipid metabolism [61]. Unlike prior findings in marmosets and humans, our results reveal exciting conservation between species, consistent with recent evidence that semen-quality traits exhibit conserved genetic regulation across mammals [62, 63]. In our study, two pathways associated with EAA were consistently identified in both the 26–59- and ≥ 60-month age groups, namely neuron projection and transmembrane transport. Notably, transmembrane transport was also enriched among the ≤25‑month‑specific hypomethylated regions, suggesting that this pathway may be involved in the epigenetic regulation of sperm biological aging across different age stages. Given its known relevance to sperm function and motility, this enrichment pattern highlights transmembrane transport as a potentially important biological process underlying variation in sperm biological age [61]. Transmembrane transport—including the movement of ions, nutrients, and other molecules across cellular membranes, particularly calcium ions—directly influences sperm motility and fertilization capacity. The pathway may therefore represent key regulatory mechanisms driving increases in epigenetic age [64].

## Conclusions

We developed a highly accurate epigenetic clock for bull sperm cells, offering a novel tool to assess the biological age of sperm and its potential implications for semen quality and fertility. Our analysis identified significant correlations between EAA and key semen quality traits, including sperm motility, semen abnormality rate, and testicular circumference. Notably, we identified critical molecular pathways linked to sperm aging, with transmembrane transport pathways playing a central role in maintaining sperm function. The cross-species human-bull sperm cell epigenetic clock offers new opportunities for comparative studies on paternal aging, paving the way for a deeper understanding of reproductive aging across species. These insights not only enhance our comprehension of sperm cell biology but also hold practical potential for improving reproductive management and extending livestock productivity.

## Supplementary Information

Additional file 1: Table S1. Metadata of bull sperm samples included in RRBS and WGBS analyses. Table S2. Information of bisulfite sequencing reads mapping. Table S3. Small-scale DNA methylation clock of sperm cells from 35 Holstein bulls. Table S4. DNA methylation clocks of Holstein bull sperm under different sequencing methods and filter criteria. Table S5. Multivariate linear regression analysis of epigenetic acceleration and breeding values of different bull complex traits, including reproductive traits. Table S6. Multivariate linear regression analysis of epigenetic acceleration and breeding values of different bull conformation traits. Table S7. Cross-species epigenetic clocks of sperm in humans and cattle. Table S8. Cross-breed epigenetic clocks of sperm in Holstein and Montbéliarde bull.

Additional file 2: Fig. S1. Workflow for the development of DNA methylation clocks in this study. Fig. S2. Global features of DNA methylation changes in Holstein bull sperm cells with aging. Fig. S3. PCA-based clustering and filtering of sperm DNA methylomes from Holstein bulls. Fig. S4. External validation of the integrated clock and RRBS-based bull sperm methylation clocks. Fig. S5. UpSet plot showing the overlap of CpG sites selected for epigenetic clock development under different combinations of sample size and sequencing coverage thresholds. Fig. S6. Prediction of epigenetic states of bovine semen samples, with the trend line fitted using a non-linear function. Fig. S7. Cross-species and age-stage-specific epigenetic clocks of bull sperm.

## Data Availability

WGBS and RRBS datasets of this article are available in the NGDC (National Genomics Data Center) repository, via the BioProject accession numbers PRJCA042261 and PRJCA032798. External validation data for the nine Holstein WGBS bulls are available from the corresponding author upon reasonable request.

## Abbreviations

EAA: Epigenetic age acceleration
GWAS: Genome-wide association study
HMR: Hypo-methylated region
MAE: Mean absolute error
MS: Initial sperm motility
NMSP: Number of motile sperm per ejaculate
NSP: Number of sperm per ejaculate
PCA: Principal component analysis
RRBS: Reduced representation bisulfite sequencing
SC: Sperm concentration per ejaculate
SCR: Sire conception rate
TSS: Transcription start site
TTS: Transcription termination site
VE: Volume of the ejaculate
WGBS: Whole-genome bisulfite sequencing
