# Supplementary material for: DNA methylation clock in bull sperm cells reveals the epigenetic aging characteristics and impact on fertility
Source: J Anim Sci Biotechnol. 2026 Jul 2;17:136. doi: 10.1186/s40104-026-01444-5 (PMC13326238; doi:10.1186/s40104-026-01444-5)
Supplement: Supplementary file 2 — Additional file 2: Fig. S1. Workflow for the development of DNA methylation clocks in this study. Fig. S2. Global features of DNA methylation changes in Holstein bull sperm cells with aging. Fig. S3. PCA-based clustering and filtering of sperm DNA methylomes from Holstein bulls. Fig. S4. External validation of the integrated clock and RRBS-based bull sperm methylation clocks. Fig. S5. UpSet plot showing the overlap of CpG sites selected for epigenetic clock development under different combinations of sample size and sequencing coverage thresholds. Fig. S6. Prediction of epigenetic states of bovine semen samples, with the trend line fitted using a non-linear function. Fig. S7. Cross-species and age-stage-specific epigenetic clocks of bull sperm. [file 40104_2026_1444_MOESM2_ESM.docx]

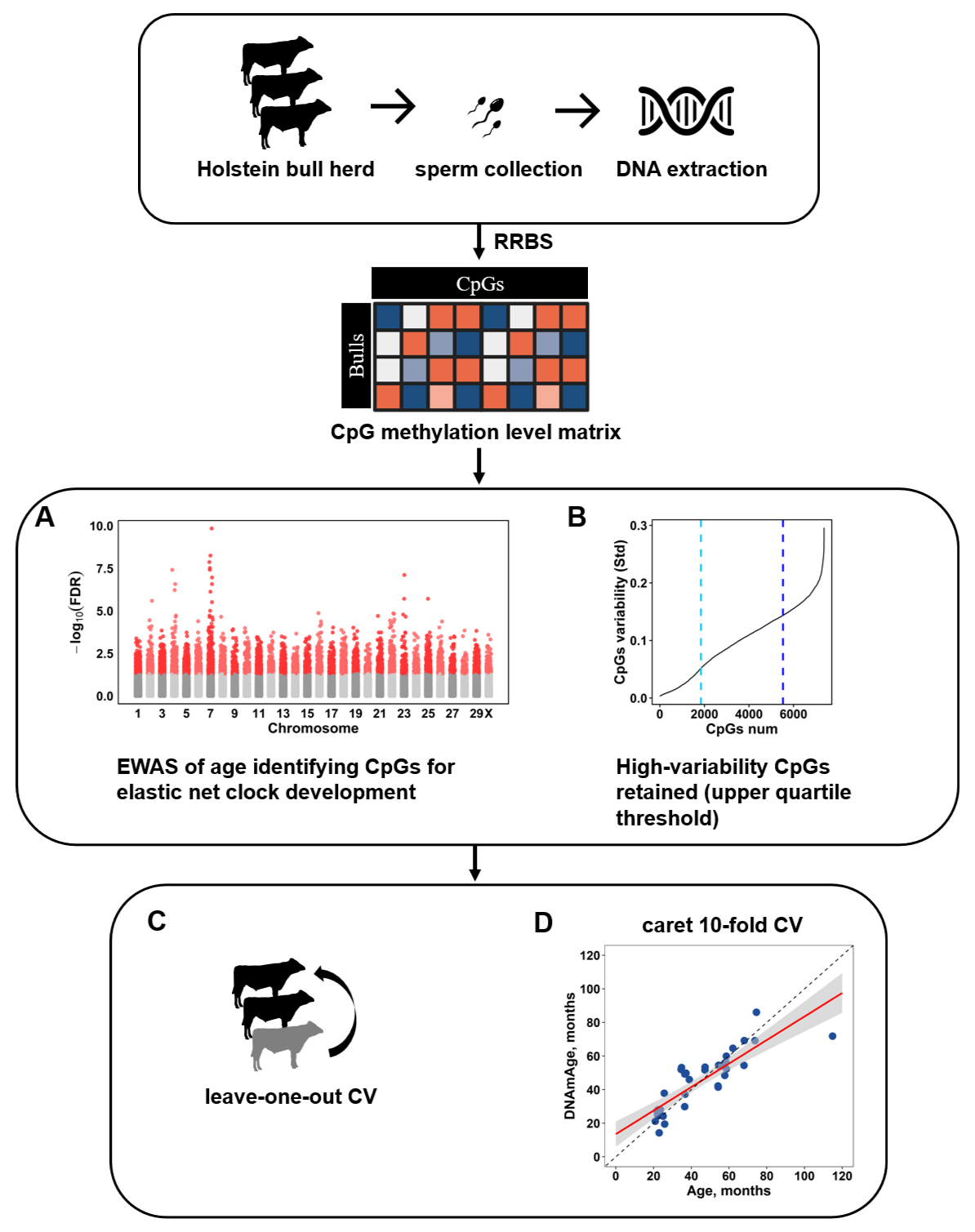


**Fig. S1** Workflow for the development of DNA methylation clocks in this study. **A** Based on a DNA methylation matrix developed from consensus CpG sites that met the predefined coverage criteria across all samples, a Manhattan plot was generated to show associations between individual CpG methylation levels and chronological age assessed using CpGassoc. CpG sites passing the Benjamini–Hochberg multiple-testing correction (FDR < 0.05) were defined as age-associated candidates. **B** Distribution of CpG methylation variability across individuals, quantified as the standard deviation of methylation levels. CpG sites within the upper quartile of the variability distribution were retained to exclude sites with extremely limited dynamic range. **C** Representative performance of sperm cell DNA methylation clocks, showing the relationship between predicted DNA methylation age and chronological age under leave-one-out cross-validation (LOOCV). The solid line indicates the regression fit, and the dashed line represents the identity line (*y* = *x*). **D** Using this unified workflow, regression analyses were performed on CpG sets obtained under different coverage depths, sequencing methods, and breeds to develop the corresponding sperm cell DNA methylation clocks. Elastic net regression models (α = 0.5) were fitted using glmnet, with penalty parameters selected by 10-fold internal cross-validation

**
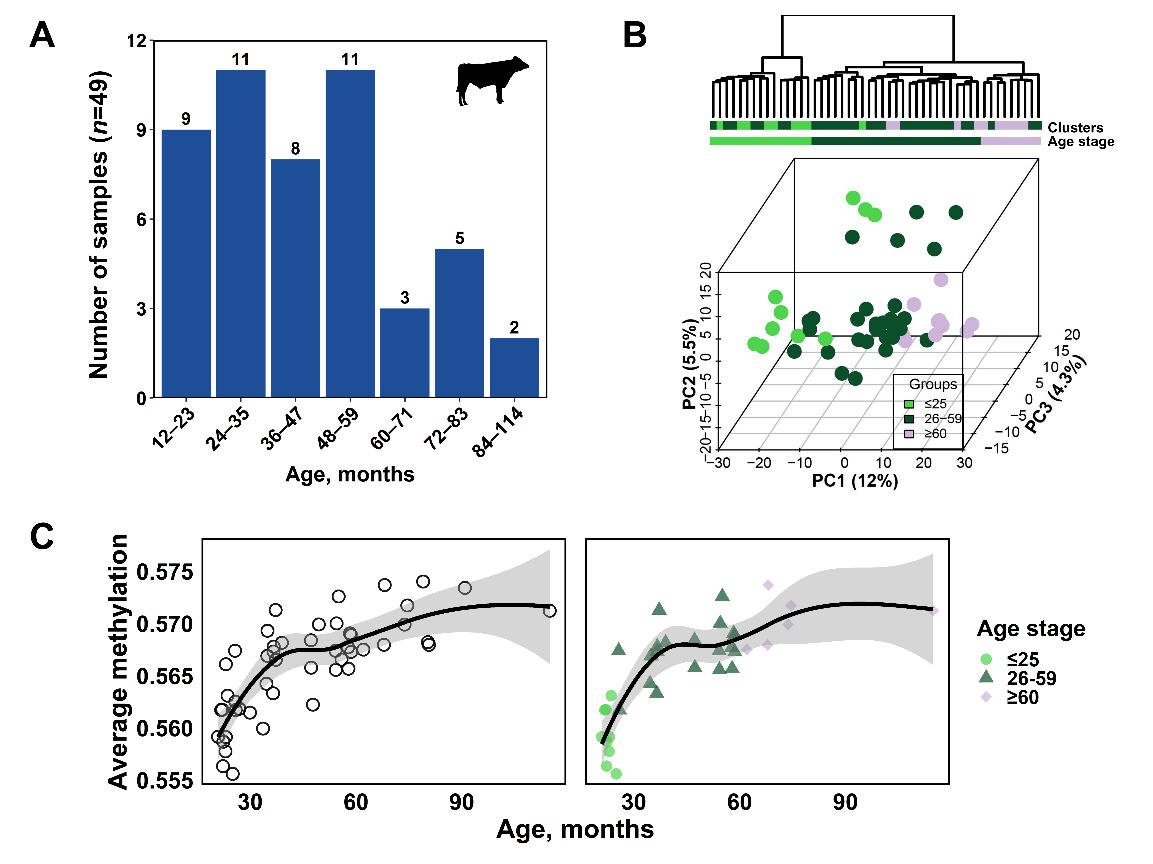
**

**Fig. S2** Global features of DNA methylation changes in Holstein bull sperm cells with aging. **A** The distribution of 49 Holstein bulls across different age stages. **B** The results of unsupervised hierarchical clustering and three-dimensional Principal Component Analysis (PCA) of the 49 RRBS samples. The dendrogram of the clustering tree displays the 49 sperm cell samples divided into three distinct clusters based on their ages. Following the clustering results, we categorized the samples into three different age stages: “≤ 25 months”, “26–59 months”, and “≥ 60 months”. **C** Age-associated increase in DNA methylation levels in bull sperm cells. Left panel: Average DNA methylation levels plotted against chronological age (months) across all 49 bull sperm samples. Each open circle represents the average methylation level of one individual sample. The black curve indicates the fitted trend, with the shaded area representing the confidence interval. Right panel: Average DNA methylation levels after excluding samples whose chronological ages were inconsistent with the defined age stages based on unsupervised hierarchical clustering results. A total of 35 samples were retained for downstream analyses. Samples are colored and shaped according to age stage: light green circles indicate bulls aged ≤ 25 months, dark green triangles represent bulls aged 26–59 months, and light pink diamonds denote bulls aged ≥ 60 months. The black curve and shaded area indicate the fitted trend and corresponding confidence interval


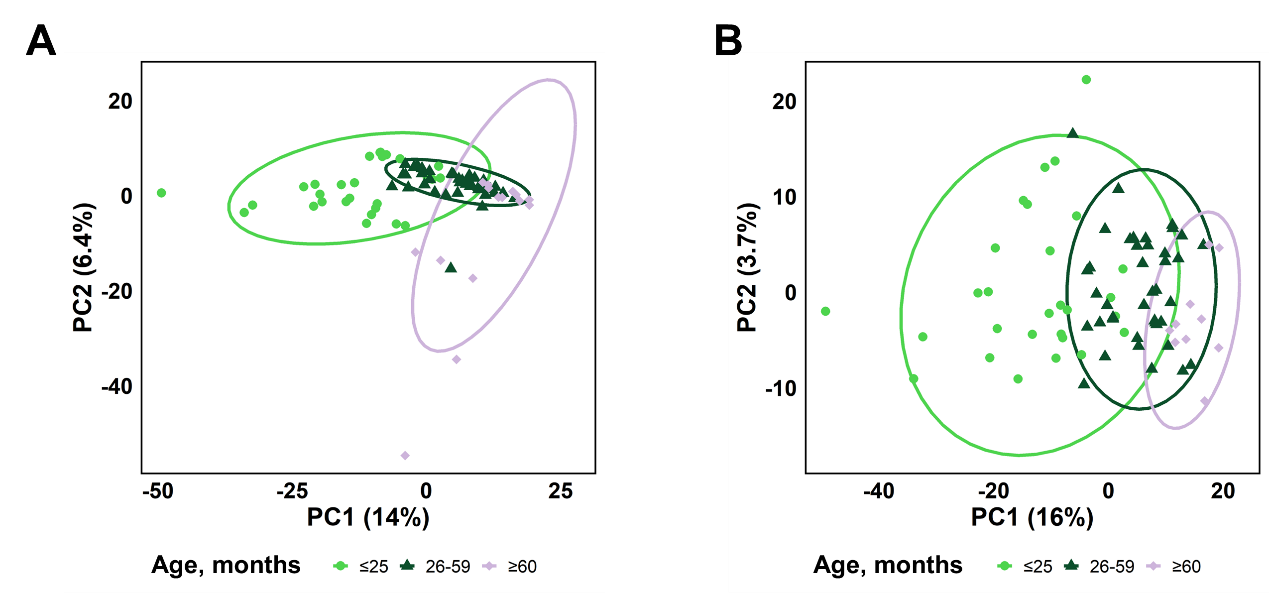


**Fig. S3** PCA-based clustering and filtering of sperm DNA methylomes from Holstein bulls. **A** PCA of sperm DNA methylomes from all 83 Holstein bulls, showing age-associated clustering patterns. **B** PCA after excluding five outlier samples identified by unsupervised hierarchical clustering, resulting in 78 retained bulls whose methylation profiles were concordant with the defined age stages. Samples are colored and shaped by age group (≤ 25, 26–59, and ≥ 60 months). Ellipses represent group-level dispersion

**

**

**Fig. S4** External validation of the integrated clock and RRBS-based bull sperm methylation clocks. **A** DNA methylation clock developed using CpG sites with a minimum coverage of ≥ 5 reads across all 49 Holstein bull sperm samples (*n* = 49), evaluated using leave-one-out cross-validation (LOOCV). **B** DNA methylation clock developed using CpG sites with a minimum coverage of ≥ 10 reads across the same 49 samples (*n* = 49), also evaluated using LOOCV. **C** External validation of the bull sperm DNA methylation clock (developed using data from 78 bulls with 10× coverage) in an independent Holstein WGBS cohort (*n* = 9)


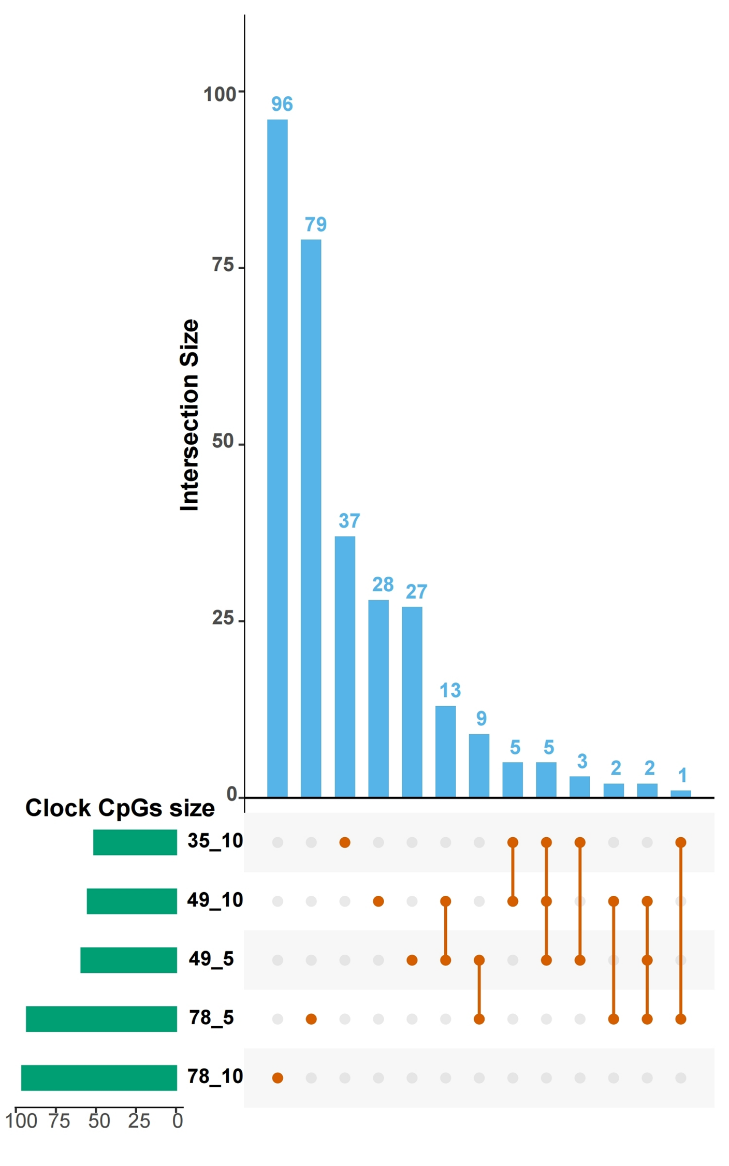


**Fig. S5** UpSet plot showing the overlap of CpG sites selected for epigenetic clock build under different combinations of sample size and sequencing coverage thresholds. Each condition is labeled in the format “number_coverage”, where the first value indicates the number of samples included in model training and the second value denotes the minimum sequencing coverage threshold applied for CpG selection


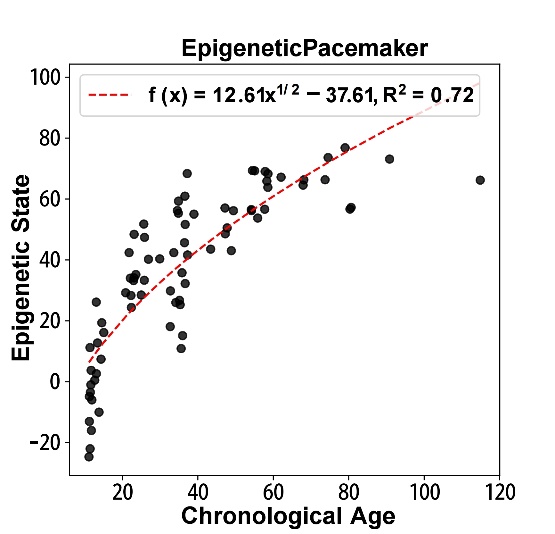


**Fig. S6** Prediction of epigenetic states of bovine semen samples (*n* = 78) using the Epigenetic Pacemaker, with the trend line fitted using a non-linear function


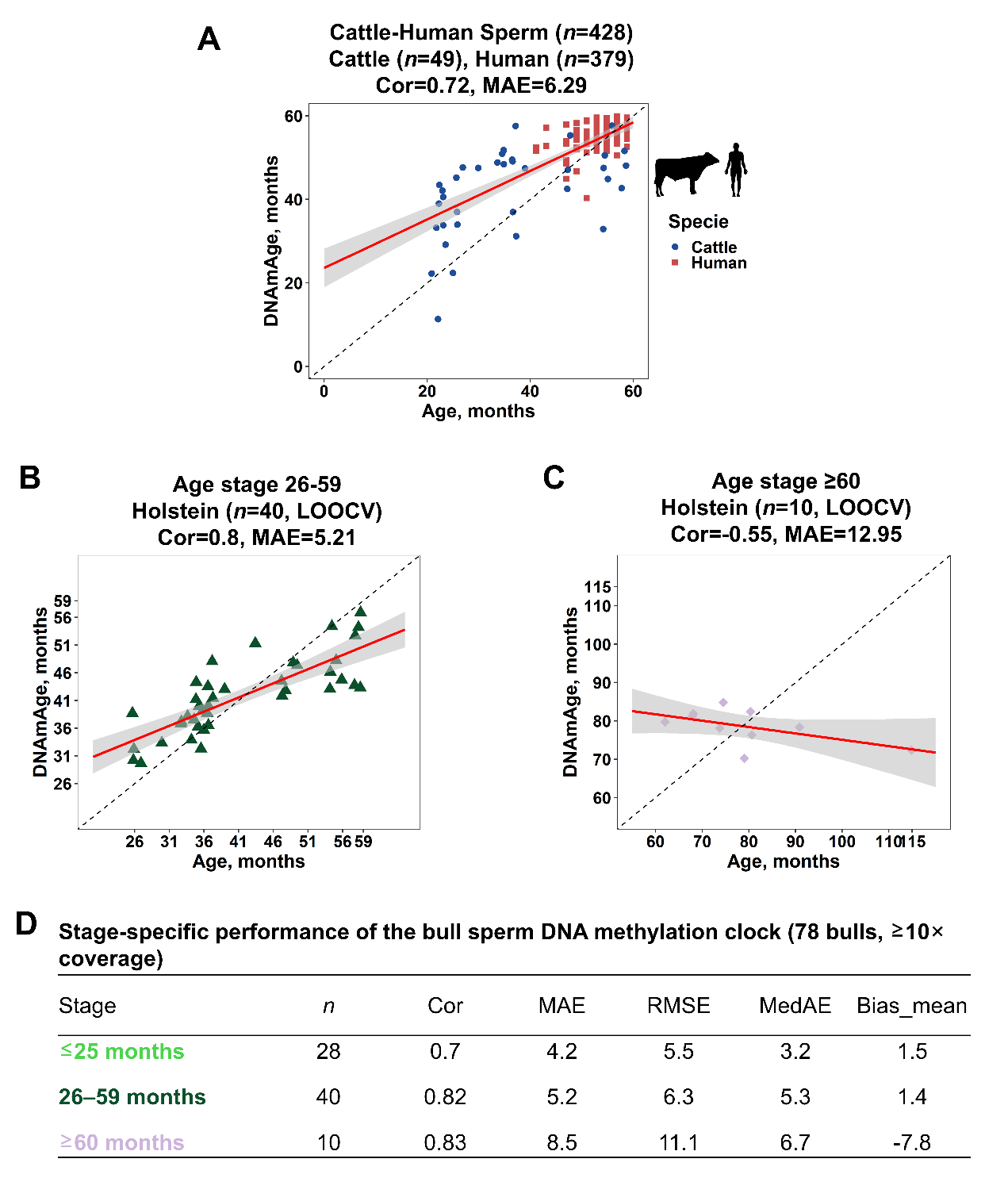


**Fig. S7** Cross-species and age-stage–specific epigenetic clocks of bull sperm. **A** Cross-species epigenetic clock developed by converting human chronological age to bovine-equivalent age (in months) based on lifespan scaling, followed by joint modeling with bovine sperm samples. Red squares represent human samples after age conversion, and black circles indicate Holstein bull samples included in the model. **B** DNA methylation clock for bull sperm samples aged 26–59 months (Holstein, *n* = 40), developed based on unsupervised hierarchical clustering and evaluated using leave-one-out cross-validation (LOOCV). **C** DNA methylation clock for bull sperm samples aged ≥ 60 months (Holstein, *n* = 10), also evaluated using LOOCV. For all panels, the dashed line indicates the line of identity (*y* = *x*), the red line represents the fitted regression line, and the shaded area denotes the confidence interval. Correlation coefficients (Cor) and mean absolute errors (MAE) are shown in each panel. *D* Stage-specific prediction accuracy of the bull sperm DNA methylation clock (78 bulls, ≥ 10× coverage)
